# Oral Contraceptives Interact with Adiposity-Associated Markers in Patients with Multiple Sclerosis

**DOI:** 10.3390/jcm13020464

**Published:** 2024-01-14

**Authors:** Véronique Ferret-Sena, Catarina Ramos, Maria João Cascais, Carlos Capela, Armando Sena

**Affiliations:** 1 Egas Moniz Center for Interdisciplinary Research (CiiEM), Egas Moniz School of Health & Science, Caparica, 2829-511 Almada, Portugal; versena@egasmoniz.edu.pt (V.F.-S.); cramos@egasmoniz.edu.pt (C.R.); 2 Nutritional Biochemistry, NOVA Medical School, Universidade Nova de Lisboa, 1150-199 Lisbon, Portugal; cascaismaria@hotmail.com; 3 Centro de Responsabilidade Integrado de Esclerose Múltipla, Hospital Santo António dos Capuchos, Centro Hospitalar Universitário Lisboa Central, EPE, 1169-050 Lisbon, Portugal; carlos.capela.trabalho@gmail.com; 4 Centro Clínico Académico de Lisboa, 1159-056 Lisbon, Portugal

**Keywords:** multiple sclerosis, adiposity, adiponectin, ageing, oral contraceptives

## Abstract

Growing evidence suggests the involvement of adipose tissue in modulating the clinical course of relapsing–remitting multiple sclerosis (RRMS). This study aimed to investigate whether the intake of combined oral contraceptives (COCs) affects body weight and leptin and adiponectin (APN) blood levels in these patients. Clinical data from 62 women (M = 33.23 year) were recorded prior to the initiation of disease-modifying therapy. Patients who were taking COCs at the time of experiencing the first symptoms of disease (COC user) were compared with those who never used these formulations or stopped taking them before disease onset (COC non-user). Bivariate Pearson’s correlations and hierarchical multiple linear regressions analysis were conducted. Normalized APN levels were lower in the COC-using patients (*p* = 0.013). Negative correlations between waist circumference and normalized APN (*p* = 0.001) were observed only in the COC non-user patients. A longer duration of COC intake was associated with increased body mass index and waist circumference (*p* = 0.003). Normalized APN predicted the MS Severity Score (MSSS) (*p* = 0.020), but this correlation was lost in the COC user patients. After adjusting for confounders, only age (*p* = 0.027) and, later, disease onset (*p* = 0.014) were correlated with the MSSS. Larger and prospective studies are needed to investigate the interactions of sex steroids with adipose metabolism in modulating disease progression.

## 1. Introduction

Multiple sclerosis (MS) is an inflammatory demyelinating and neurodegenerative disease of the central nervous system (CNS). It may be caused by a complex interplay between genetic and environmental or lifestyle risk factors [1]. Growing evidence supports the role of adiposity-related mechanisms in modulating the clinical course of the disease [2,3]. Interestingly, interactions between sex hormones and adipose metabolism have been suggested to be involved in the risk of MS [4,5]. Relapsing–remitting MS (RRMS) is more common in women. Sex hormones may play a role in gender differences associated with susceptibility and disability, as well as disease progression [6]. The use of combined oral contraceptives (COCs) may provide an opportunity to investigate the role of sex steroids in MS. Our group’s previous work suggested that COC intake may interact with lipoprotein metabolism in these patients [7]. Leptin and adiponectin (APN) are the major adipokines (hormones secreted by adipose tissue) that regulate energetic metabolism and immune function, and they have been implicated in MS and other immune-mediated diseases [2,8]. Recently, sex-specific changes in the levels of adipokines in blood were associated with disability outcomes and brain volumes in MS patients [3]. In healthy women, COC intake may alter serum lipoprotein and adipokines levels [9,10]. It was suggested that the production of free radicals by COCs or their metabolites may change the synthesis of lipoproteins, leptin, and APN [10]. However, to our knowledge, no study to date has investigated the potential effects of COC use on adipokine levels in women with MS. Therefore, the aim of this exploratory work was to investigate whether COC use affects adiposity markers in RRMS patients and their associations with the clinical course of the disease.

## 2. Materials and Methods

### 2.1. Patients

The studied population included 62 women diagnosed with relapsing–remitting MS (RRMS) according to the 2017 revised McDonald criteria [11] and followed at the MS outpatient clinic of a university hospital in Lisbon (Portugal). This cohort was part of a larger population previously investigated by our group [7] and included all patients in whom leptin and APN analysis were available before DMT initiation.

This study was approved by the Ethics Committee of the University Centro Hospitalar de Lisboa Central (Lisbon, Portugal). All patients gave written informed consent, in accordance with the Declaration of Helsinki.

Demographic and clinical information, as well as blood samples at a stable phase of the disease, were collected by visiting the hospital. Disease onset (DO) was defined as the age at which the first symptoms suggestive of MS appeared. The Expanded Disability Status Scale (EDSS) score was determined and converted to the MS Severity Score (MSSS) [12]. The number of relapses within the last year prior to enrolment were recorded. Relapses were defined as the appearance or worsening of neurological signs lasting over 24 h and without the presence of fever. Patients with an associated hormonal or metabolic disorder, those taking lipid-lowering agents, and those who received steroid therapy or had a clinical relapse within previous 4 weeks were excluded. Menopausal, pregnant, and breastfeeding women, as well as those who had a delivery in the last 6 months, were also excluded. Data regarding BMI measurements (calculated as weight in kilograms divided by height in metres squared), waist and hip circumferences, and patients’ history of smoking habit and COC intake were collected. Women labelled smokers reported smoking regularly (at least five cigarettes per day) at the time of the clinical and laboratory evaluations. Women were classified into the COC non-user group if they had never consumed a COC or discontinued its intake before DO (COC−) or and the COC user group if they had initiated pill use before DO and maintained intake at the time of clinical evaluation and biochemical analysis (COC+).

### 2.2. Biochemical Analysis

Blood samples were collected under fasting conditions shortly after the day of clinical data collection and neurological examination. Serum samples were stored at −80 °C, and biochemical measurements were performed blindly with respect to the participants. Enzyme-linked immunosorbent (ELISA) assays were used for measurements of adiponectin (Mercodia, Uppsala, Sweden, eBioscience, San Diego, CA, USA) and leptin (R&D Systems, Minneapolis, MN, USA). Leptin and adiponectin levels in serum were normalized for BMI (i.e., adipocytokine concentrations were divided by BMI).

### 2.3. Statistical Methods

Means (standard deviations (SDs) and medians (inter-quartile ranges (IQRs)) were used to describe demographic, clinical, and laboratory data. The results were pairwise-compared between the COC− and COC+ subgroups using two-sided Mann–Whitney non-parametric tests for continuous data. Bivariate Pearson’s correlations between the adiposity-related markers were analysed. Several hierarchical multiple linear regressions were performed using the enter method to assess the effect of COC intake on the associations between adiposity and the clinical variables. The clinical data were inserted as dependent variables at step 1; COC use was inserted at step 2, and age, disease duration, and number of relapses were inserted at step 3. Durbin–Watson statistics were calculated to assess the presence of autocorrelations in the residuals using scores of approximately 2. Multicollinearity was checked using a variance inflation factor (VIF) value of 5. All statistical analyses were performed considering a significance level of 0.05 (α < 0.05) and were developed using the Statistical Package for Social Sciences (IBM SPSS Statistics, version 28.0, IBM Corp, Armonk, NY, USA).

## 3. Results

The main demographic, clinical, and laboratory characteristics of the whole cohort, stratified according to COC use, are presented in Table 1.

Both subgroups of subjects had a mean disease duration of approximately 4 years. The mean duration of COC use was 10.94 years (SD = 6.53). The composition of COC formulations was available in 78% of patients. All women took formulations containing ethinyl oestradiol (EE) combined with different progestins, with desogestrel or gestodene being the two most common. These groups were quite small, and no differences based on the dose of EE or the type of progestin content could be evaluated. Obesity (BMI ≥ 30) was only observed in eight patients. The EDSS (U = 254, *p* = 0.001) and MSSS values (U = 247, *p* = 0.001), mean APN (U = 197.5, *p* = 0.016), and normalized APN levels (U = 193.5, *p* = 0.013) were lower in COC users. Pearson’s bivariate correlations between adiposity variables found positive correlations between BMI and leptin and waist and leptin (r = 0.777 *p* < 0.001) in the global sample and both subgroups. In contrast, significant negative correlations between waist circumference and APN (r = −0.423, *p* = 0.040) and normalized APN values (r = −0.621, *p* = 0.001) were only found in patients who did not use COC. Patients who used COCs for more than 10 years had higher BMI (M = 27.31; SD = 5.13) (t(30) = 3.208, *p* = 0.003) and waist circumference values (M = 81.20; SD = 8.89) (t(27) = −2.363, *p* = 0.003)), but no significant differences in APN levels compared to those who used COCs for 10 years or less were found. No significant associations were observed between smoking and the adiposity markers or the clinical variables.

To assess which adiposity variables (Step 1) predict clinical variables and the effects of COC intake (Step 2) on these relationships, several hierarchical regressions were conducted. All assumptions were confirmed in each model for each dependent variable. The regression model with the MSSS as the dependent variable showed that the model in step 1 was significant (*F* (4.44) = 3.448, *p* = 0.016; R^2^_adj_ = 0.175). Normalized APN was the only significant variable (β = 0.355, *t* = 2.419, *p* = 0.020). In step 2, normalized APN lost significance, and COC remained significant (β = 0.431, *t* = −3.093, *p* = 0.004) in a model that was also significant (*F* (5.41) = 5.234, *p* ≤ 0.001, R^2^_adj_ = 0.315). In step 3, the model was adjusted for age, disease duration, and number of relapses. The COC variable remained the only significant predictor of MSSS. Age (β = 0.335, *t* = 2.365, *p* = 0.005) and disease duration (β = −0.318, *t* = −2.400, *p* = 0.021) were also found to be predictors of MSSS. The regression model with the number of relapses as the dependent variable did not yield significant results in step 1. In step 2, normalized APN was a significant predictor of a lower number of relapses (β = −0.373, *t* = −2.173, *p* = 0.036) but the model explained only 0.9% of the explained variance of the number of relapses and was not significant (*F* (5.41) = 1.913, *p* = 0.113). In step 3, the model was adjusted for age and disease duration, and the APN/BMI remained the only significant predictor of the number of relapses. Table 2 displays steps 1, 2, and 3 for each of the hierarchical linear models. We repeated the hierarchical multiple regression with the same variables while controlling for the effects of age, disease onset, and smoking. The models showed an increase in the explained variance of the dependent variables, but normalized APN and COC lost significance. In the model with the MSSS as the dependent variable, age (β = −1.367, *p* = 0.027) and age at disease onset (β = 1.653, *p* = 0.014) were the only significant ones.

## 4. Discussion

Our study suggests that COC use is associated with decreased serum APN and normalized APN levels in RRMS patients. In contrast, no differences in BMI, waist circumference, waist/hip ratio, and serum leptin levels were found between COC user and non-user patients. Similar results were reported in healthy women, although the effects of COCs on APN levels may be influenced by the progestin content of COC preparations [9,10]. Androgens inhibit APN production, contributing to the lower levels of this adipokine observed in men compared to women. The most important factor regulating APN is adipose tissue. Lower blood levels of this adipokine are generally observed in obesity and associated comorbidities. In contrast, in MS and other immune-mediated or autoimmune diseases, increased circulating levels of APN are frequently found [2,8].

This scenario indicates that, in inflammatory pathologies, there may be alterations in the physiological body fat mechanisms regulating the production or clearance of APN. It is worth noting that in subjects undergoing abdominal surgery, APN secretion was negatively correlated with BMI, visceral adipose tissue, and waist circumference exclusively in women [13]. We observed negative correlations between APN and waist circumference solely in the COC non-user patients. Furthermore, a longer duration of COC intake was found to be associated with higher levels of BMI and waist circumference, without significantly changing APN concentration. These findings suggest that COC formulations or their metabolites may affect adipose metabolism regulating circulating APN levels in RRMS patients.

Reports on the pathophysiological role of APN blood levels in MS are conflicting. While some studies did not find any significant effect of APN on the risk [14,15] and the clinical course of the disease [16,17], most authors have associated this adipokine with the inflammatory activity and severity of MS [3,18,19,20,21]. The effects of APN on the activity of the disease were found to be independent of BMI [3,21] and to involve sex-dependent mechanisms [3,19]. Moreover, Bove et al. (2016) [22] also observed sex-based differences in the association of BMI with disease severity. Nevertheless, none of the aforementioned studies included data concerning COC behaviour. In line with recent works [23,24], our findings suggest no influences on relapse risk associated with COC use. In contrast, lower disability scores, APN, and normalized APN levels were observed in COC users. This scenario could be due to the milder natural course of the disease in these patients, promoting COC intake. Although selection bias cannot be excluded, this hypothesis seems unlikely to explain our results. Despite ample evidence of the anti-inflammatory proprieties of APN, this adipokine has also been suggested to have pro-inflammatory effects in MS and other autoimmune diseases [2,8]. Our findings support a potential dual role for APN in RRMS patients, independent of BMI. However, the associations of normalized APN with the number of relapses and disability scores were found to depend on concomitant COC use.

Taken together, these data suggest that COC intake may represent an important confounding factor in studies investigating the effects of this adipokine on the clinical course of RRMS. Nevertheless, after adjusting for confounders, in line with a prospective study by Otero-Romero et al. [24], no effects of COC use on disability accrual were observed. Only age and, later, onset disease remained significantly correlated with MSSS. These data indicate that the potential role of adipose tissue in the disease may vary with age. Interestingly, Foong et al. [25] observed that ageing decreases the effects of physical activity on adiposity. Our results support the current evidence implicating ageing-related mechanisms in promoting disability progression since the earliest stages of MS [26,27]. 

This study has several limitations due to its cross-sectional design and small sample of participants. We were unable to investigate the potential effect of different COC formulations, which may influence the susceptibility for MS [28] and APN levels [9,10]. Circulating APN oligomers, which exhibit different pro- or anti-inflammatory proprieties in MS [20,21], were not analysed. The potential effect of sex steroids in the production or clearance of the different APN oligomers in these patients deserve further investigation. Information concerning physical activity and diet were not recorded, and these factors could affect APN blood levels [8]. However, the influence of these potential confounders seems unlikely, as most patients had a BMI in the normal range and low motor disability scores. Additionally, all patients were enrolled before the initiation of disease-modifying therapy (DMT), a factor that has not been controlled by most previous studies and could change adipokine levels [7,17].

In conclusion, our findings suggest that COC use decreases APN blood levels in women with RRMS independently of BMI. They also suggest that the potential effects of COCs and adiposity in the pathophysiology of the disease are influenced by age-related mechanisms. Larger studies, specifically prospective studies, are needed to investigate the interactions between sex hormones and adipose tissue in modulating the clinical course of MS. 

## Figures and Tables

**Table 1:** Clinical and demographic characteristics and adiposity-associated markers of the cohort, stratified according to combined oral contraceptive use.

Characteristics	COC− (*n* = 30)		COC+ (*n* = 32)		*p*-Value
	M (SD)	Median (IQR)	M (SD)	Median (IQR)	
Age (years)	34.70 (8.47)	35.00 (12)	31.84 (6.53)	31.00 (10)	0.109
Disease onset (years)	30.10 (8.43)	30.00 (13)	27.16 (6.21)	27.00 (9)	0.150
Relapses (*n*, %) ^a^	27 (90%)	-----	29 (90.6%)	-----	0.667
MSSS	4.00 (2.46)	3.62 (3.86)	2.04 (1.72)	1.61(2.14)	**0.001**
EDSS	2.00 (1.20)	2.00 (2.4)	1.03 (0.80)	1.00 (1.0)	**0.001**
BMI	23.33 (11.72)	23.00 (7)	24.81 (5.03)	24.50 (9)	0.241
Waist circumference (cm)	78.70 (11.72)	79.00 (21)	76.72 (11.39)	77.00 (18)	0.599
Waist/hip	0.78 (0.07)	0.80 (0.10)	0.77 (0.08)	0.76 (0.11)	0.317
Leptin (ng/mL)	19.27 (14.05)	15.00 (22.10)	21.17 (14.54)	20.35 (25.2)	0.375
Leptin/BMI	0.78 (0.47)	0.68 (0.90)	0.81 (0.42)	0.74 (0.69)	0.499
APN (µg/mL)	10.26 (3.81)	10.10 (5.65)	7.93 (3.26)	7.50 (2.70)	**0.016**
APN/BMI	0.45 (0.20)	0.45 (0.26)	0.33 (0.14)	0.34 (0.08)	**0.013**
Smokers (*n*, %) ^a^	10 (33.3%)	-----	12 (37.5%)	-----	0.732

Continuous variables were compared using the Mann–Whitney non-parametric test. Categorical variables are expressed as frequency n (%). ^a^ A chi-square test was used to compare means for the number of relapses within the previous year to sampling and the number of smokers. COC+, combined oral contraceptive user patients; COC−, combined oral contraceptive non-user patients; MSSS, Multiple Sclerosis Severity Score; EDSS, Expanded Disability Status Scale; BMI, body mass index; APN, adiponectin. Bold values mean significant differences.

**Table 2:** Hierarchical linear models used to assess the associations between adiposity markers and clinical variables.

Variables	β	*t*	*p*	*F* (*df*)	*p*	*R* ^2^ *_ajust_*
**Model 1 (MSSS as dependent variable)**						
Step 1				3.448 (4.2)	**0.016**	0.175
Leptin/BMI	0.274	1.682	0.100			
APN/BMI	0.355	2.419	**0.020**			
Waist circumference	0.188	0.841	0.405			
Waist/hip	0.183	0.950	0.347			
Step 2				5.234 (5.41)	**≤0.001**	0.315
Leptin/BMI	0.240	1.610	0.115			
APN/BMI	0.154	1.032	0.308			
Waist circumference	0.136	0.665	0.510			
Waist/hip	0.077	0.428	0.671			
COC	−0.431	−3.093	**0.004**			
Step 3				4.792 (8.38)	**≤0.001**	0.397
Leptin/BMI	0.194	1.321	0.194			
APN/BMI	0.051	0.338	0.737			
Waist circumference	0.018	0.090	0.928			
Waist/hip	0.070	0.411	0.683			
COC	−0.391	−2.951	**0.005**			
Age	0.335	2.365	**0.023**			
Disease Duration	−0.318	−2.400	**0.021**			
Number of relapses	−0.043	−0.330	0.743			
Model 2 (number of relapses as dependent variable) *						
Step 1				2.426 (4.42)	0.063	0.110
Leptin/BMI	0.159	0.938	0.353			
APN/BMI	−0.353	−2.310	**0.026**			
Waist circumference	0.060	0.257	0.798			
Waist/hip	−0.179	−0.894	0.377			
Step 2				1.913 (5.41)	0.113	0.090
Leptin/BMI	0.155	0.905	0.371			
APN/BMI	−0.373	−2.173	**0.036**			
Waist circumference	0.055	0.231	0.818			
Waist/hip	−0.190	−0.920	0.363			
COC	−0.044	−0.272	0.787			
Step 3				1.799 (7.39)	0.115	0.108
Leptin/BMI	0.074	0.414	0.681			
APN/BMI	−0.372	−2.143	**0.038**			
Waist circumference	0.134	0.550	0.585			
Waist/hip	−0.158	−0.767	0.448			
COC	−0.067	−0.414	0.681			
Age	−0.169	−0.992	0.327			
Disease Duration	−0.143	−0.894	0.377			

Results were obtained from the hierarchical multiple linear regression tests using a stepwise method. All assumptions were confirmed (i.e., Durbin–Watson and VIF. β = standardized beta; *t* = t-student; *F* = F-test; *df* = degrees of freedom; *R*^2^*_ajust_ =* adjusted R square; MSSS, Multiple Sclerosis Severity Score. * Number of relapses within the previous year to sampling. COC, combined oral contraceptive; BMI, body mass index; APN, adiponectin. Bold values mean significant values.

## Data Availability

Data pertaining to this manuscript can be made available upon request to the corresponding author.
